# Sparse Spatial-Temporal Emotion Graph Convolutional Network for Video Emotion Recognition

**DOI:** 10.1155/2022/3518879

**Published:** 2022-09-28

**Authors:** Xiaodong Liu, Huating Xu, Miao Wang

**Affiliations:** School of Software, Henan University of Engineering, Zhengzhou, China

## Abstract

Video emotion recognition has attracted increasing attention. Most existing approaches are based on the spatial features extracted from video frames. The context information and their relationships in videos are often ignored. Thus, the performance of existing approaches is restricted. In this study, we propose a sparse spatial-temporal emotion graph convolutional network-based video emotion recognition method (SE-GCN). For the spatial graph, the emotional relationship between any two emotion proposal regions is first calculated and the sparse spatial graph is constructed according to the emotional relationship. For the temporal graph, the emotional information contained in each emotion proposal region is first analyzed and the sparse temporal graph is constructed by using the emotion proposal regions with rich emotional cues. Then, the reasoning features of the emotional relationship are obtained by the spatial-temporal GCN. Finally, the features of the emotion proposal regions and the spatial-temporal relationship features are fused to recognize the video emotion. Extensive experiments are conducted on four challenging benchmark datasets, that is, MHED, HEIV, VideoEmotion-8, and Ekman-6. The experimental results demonstrate that the proposed method achieves state-of-the-art performance.

## 1. Introduction

With the rapid development of the Internet and mobile devices, people's demand for media content understanding has become more and more extensive. At present, researchers have made great progress in video content understanding such as video action recognition and detection. However, the research on video emotion recognition is relatively limited. Video emotion recognition is an important part of video understanding. In recent years, video emotion recognition has attracted more and more attention because of its potential applications in advertising, psychological barrier understanding, video summarization, action recognition [1], object recognition [2], anomaly detection [3], real-time object detection and tracking [4], image white balance [5], and other fields. Compared with video understanding such as action recognition [6] and detection, in addition to being affected by multiple factors such as lighting conditions, various perspectives, and composite backgrounds, video emotion recognition also faces the following two major challenges. One is the diversity of emotion expression; people's face, body, scene, and surrounding environment all express certain emotions. The other is the relevance of emotional expression. The faces, scenes, and surrounding environment in the video are interrelated. Therefore, how to recognize emotion from the video is a challenging topic.

The early research studies on video emotion recognition mainly used low-level manual features. Kang [7] selects motion and color features to detect emotional events based on the Hidden Markov model. Due to the limited representation ability of low-level features, some researchers use attribute features. The image is represented as the response diagram of multiple pretrained detectors. For example, SentiBank [8] uses the response scores of 1200 concept detectors to represent images.

In recent years, with the success of convolutional neural networks (CNN) in action recognition and object recognition [9], some researchers have tried to use CNN to improve the accuracy of emotion recognition. Different from image recognition, because human emotions are expressed in many ways, such as face and voice, video emotion recognition adopts the features of multiple modes to improve recognition accuracy. Kaya et al. [10] use a least squares regression classifier and weighted score fusion to fuse appearance features and audio features. In addition to appearance and audio, the human body, action, and environment also contain certain emotional clues. The fusion of these emotional clues can further improve the performance of emotion recognition. Jiang et al. [11] propose an emotional context fusion network, which first extracts the event, object, and scene features in the video and then fuses these features by the context fusion network to improve the accuracy of video emotion recognition.

However, these features are independent of each other, and the potential relation between multimodal data in the video has not been considered. To solve this problem, Xue et al. [12] propose a Bayesian nonparametric multimodal data modeling architecture to learn emotions in the video. The CNN is used to extract the appearance features of keyframes and the Mel frequency cepstrum coefficient is used as audio features, and then, the hierarchical Dirichlet process is used to mine their potential emotional events. This research correlates different modes and establishes the relationship between different information, but it does not reason about the relationship between information.

In recent years, graph convolutional network (GCN) [13] based on relational reasoning has attracted more and more attention. It has been used in many fields, such as video action recognition and large-scale object detection. In the research of emotion recognition, Huadong Ma et al. [14] construct an emotion graph using emotion context elements and reasons about the emotion relationship according to the graph convolution network. It models humans and their context in a single image. Although GCN-based relational reasoning has been widely used in the fields of video action recognition [15] and large-scale object detection [16], these methods cannot be directly applied to video emotion recognition. Although the research on video emotion recognition has made great progress, it still has three major challenges.

**Figure 1:**
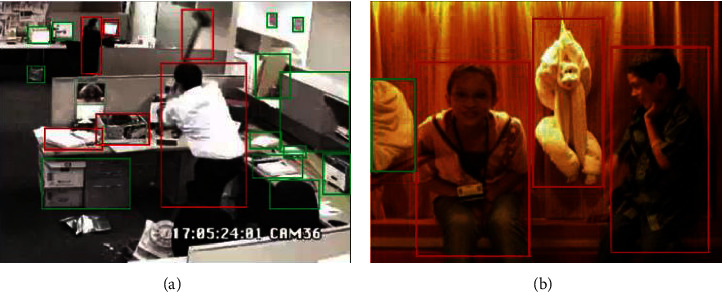
The motivation of emotional relationship graph.

1. The existing graph-based methods propagate features between all edges. For example, in group action recognition [17], edges between all vertices are established and their relationships are reasoned about to recognize group action. However, this fully connected graph has some disadvantages in video emotion recognition. Firstly, there are many backgrounds or negative samples in emotion proposal regions. If the full connection matrix is used to establish the relationship between background or negative samples, these redundant edges will lead to more computation and redundant information. It is difficult to ensure that the graph structure can focus on the most relevant relationship to identify the emotion. For example, let us try to build the emotional relationship of the video frame, as shown in Figure 1. In Figure 1(a), we can easily judge that the emotional category of the video is “angry” by reasoning about the relationship between the red emotional proposal regions. Building the emotional graph with all the emotional proposal regions not only increases the amount of computation but also brings a lot of redundant information. Therefore, how to construct a sparse spatial emotion graph is a challenge for video emotion recognition.
2. In the construction of the temporal graph, the time evolution of all nodes is constructed in the existing graph-based method. For example, in skeleton-based action recognition, the temporal relationship of all joints is established [15]. Similar to the spatial graph, building connections for all nodes will also produce more computation and redundant information. It is difficult to ensure that the graph structure can focus on the most relevant relationships to identify the emotion.
3. Due to the diversity of emotional expression, the human's face, body, scene, and surrounding environment all express certain emotional clues, but they contain different amounts of emotional information. Therefore, how to use rich emotional cues, simultaneously, without losing the robustness of fewer emotional cues, is another challenge for video emotion recognition.

To overcome the above challenges, this study proposes a sparse spatial-temporal emotion graph convolutional network-based video emotion recognition method (SE-GCN). Firstly, the emotion proposal regions are extracted by the region proposal network (RPN). The *d*-dimensional appearance features of each emotion proposal region are obtained by the feature extract network. Secondly, the sparse spatial-temporal emotion graph is constructed according to these original features. For the spatial graph, the emotional relationship between any two emotion proposal regions is calculated by the emotional relationship recognition module. The sparse spatial graph is constructed according to the emotional relationship. For the temporal graph, the emotional cue analysis module is used to analyze the emotional information contained in each emotion proposal region, and the sparse temporal graph is constructed by using the emotion proposal regions with rich emotional cues. Thirdly, the reasoning features of emotion relationships are obtained by the spatial-temporal GCN. Finally, the features of the emotion proposal regions and the spatial-temporal relationship features are fused to recognize the video emotion.

The main contributions of this study are as follows:

- We propose a sparse spatial-temporal emotion graph convolutional network-based video emotion recognition method (SE-GCN), which is established to reason about the emotional relationship of selected emotion regions.
- A sparse spatial emotion graph is designed to reduce the amount of computation and redundant information. The graph structure focuses on the most relevant relationship to improve the accuracy of emotion recognition.
- A sparse temporal emotion graph is designed, and the emotion proposal regions with rich emotion clues are selected to construct the graph structure. The time evolution mainly focuses on the elements with rich emotional clues, which reduce the amount of computation and redundant information.
- To make full use of the rich emotional cues and simultaneously without losing the robustness of fewer emotional cues, the emotion score of each proposal region is obtained based on the attention mechanism and the features of proposal regions are aggregated according to the emotion score.

## 2. Related Work

### 2.1. Video Emotion Recognition

Humans express emotion through multiple modes such as face and voice. Therefore, some researchers fuse the features of multiple modes to recognize video emotion. Multimodal emotion recognition learns emotion feature from multiple modes and then fuse these features. It mainly includes two methods: decision-level fusion and feature-level fusion. The decision-level fusion models the input of different modes, and the features of different modes are fused at the decision-making level. Kim et al. [18] use the average value of the upper part of the face, the lower part of the face, and audio to obtain the final emotion label. Kaya et al. [10] use a least squares regression classifier and weighted features fusion to fuse appearance features and audio features. The feature level fusion first fuses the multimodal features into a feature vector and then uses the fused feature vector for emotion classification. It can take advantage of the complementarity between the multimodal features. For example, Tzirakis et al. [19] propose a fusion method that fuses audio and appearance features. Firstly, the 1280-dimensional audio feature is extracted by the convolution neural network, and the 2048-dimensional appearance feature is extracted by the 50 layers depth residual network. Then, the two features are connected into a 3328-dimensional feature vector, which is fed into a two-layer recurrent neural network with 256 neurons. The decision-level fusion and the feature-level fusion both adopt supervised learning. In terms of unsupervised learning, Xu et al. [20, 21] transfer knowledge from auxiliary image and text data to better understand the video content. These multimodal emotion recognition algorithms treat video frames equally and cannot focus on the key video frames. However, due to the sparsity of emotional expression in the video, human emotion can only be recognized at some specific time in long-term expression. The keyframes are the most relevant expression of emotion in the video. In video emotion recognition, we can focus on key video frames to reduce redundant information, so as to improve the accuracy of video emotion recognition.

There are mainly two methods to solve the sparsity of emotional expression. One is the keyframes extraction algorithm. Fatemeh et al. [22] propose a multimodal emotion recognition system that is based on audio and appearance features. The video is summarized in a set of keyframes. Each frame extracts Mel frequency cepstrum coefficients from audio and the appearance features by the geometric relationship of facial feature points. Singh et al. [23] select keyframes based on facial action units and locate the key segments in audio by the synchronous and asynchronous time relationship. Zhang and Zu [24] transform frame-level spatial features into another feature space by kernel function to capture higher-level distinctive emotional features. They also use denoising to reduce the influence of noise in the video, which further improves recognition accuracy and computational efficiency. The other method adopts an attention mechanism. Barros et al. [25] implement a deep convolutional neural network architecture to learn the location of emotional expression in chaotic scenes. A series of experiments are conducted to detect regions of interest based on emotional stimuli. The results show that the attention mechanism can improve the performance of emotional recognition. Wang et al. [26] propose two-stage attention and two-stage multitasking learning framework. In the first stage, the attention mechanism is used to automatically extract and enhance the features of the corresponding region. Next, the recurrent neural network and self-attention network are used to adaptively use the relationship of different levels. Zhang et al. [27] perceive which time-frequency region of the speech spectrum is more related to emotion expression by the attention mechanism. Lee et al. [28] selectively focus on the emotion-rich part of facial videos according to a spatiotemporal attention network.

However, the connection between features of different modes is not established, which limits its performance to a certain extent. To capture the statistical interaction between modes, Wang et al. [29] propose a multimodal domain adaptive method to obtain the interaction between modes. Some researchers establish the relationship between modes based on canonical correlation analysis. Sarvestani et al. [30] propose a feature fusion method based on sparse kernel probabilistic canonical correlation analysis, which unifies the potential variables of the two modes into a feature vector with acceptable size. Qiu et al. [31] use the depth canonical correlation analysis method to learn a nonlinear feature transformation from two modes to a highly correlated space.

However, these studies only establish the relationship between modes and have not yet conducted relational reasoning on the emotional elements in the video. Zhang et al. [14] construct an emotion graph using context elements and reason about the emotion relationship by the graph convolution network. The emotion graph mainly models the person and context in a single image, and the relationship of all emotional elements is built, which has redundant edge information. In our sparse spatial-temporal graph, the spatial graph only selects proposal regions with close emotional relationships to build edges to reduce the redundant information and the computation. The temporal graph focuses on the emotional elements with rich emotional clues.

### 2.2. Graph-Based Relational Reasoning

In recent years, graph-based relational reasoning has attracted more and more attention. GCN [13] extends the convolutional neural network to the graph structure. Wang et al. [32] capture the relationship between objects by GCN. Wu et al. [17] establish a flexible and efficient person relationship graph to capture the appearance and location relationship between persons. It learns the potential relationship between persons by the graph convolution operation to recognize the group action in the video. These studies establish a full connection graph between all vertices and do not impose constraints on the sparsity of the graph. However, there may be background or negative samples in the candidate box. These redundant edges will lead to greater computational cost, and the propagation between vertices may be invalid or even wrong. To solve this problem, for each region candidate box, Xu et al. [16] only select *t* maximum values per row in the adjacency matrix. The value in the adjacency matrix reflects the correlation between candidate regions to some extent, but this judgment method is not very accurate. Velickovic et al. [33] propose a graph attention network, which purpose is to use the self-attention layer to classify the nodes in the graph and focus on those nodes which play a greater role. Li et al. [34] introduce an encoder-decoder structure to capture the potential dependencies of specific actions. Inspired by the above work, a sparse spatial-temporal graph is constructed. For the spatial graph, the emotional relationship between any two emotion proposal regions is calculated and the sparse spatial graph is constructed according to the emotional relationship. For the temporal graph, the emotional information contained in each emotion proposal region is first analyzed and the sparse temporal graph is constructed by using the emotion proposal regions with rich emotional cues.

## 3. Sparse Emotion Graph Convolutional Network

This section will introduce the sparse emotion spatial-temporal graph convolutional network-based video emotion recognition method (SE-GCN) in detail. We first introduce the system architecture, and then, each module of the system will be introduced in detail.

### 3.1. System Framework

**Figure 2:**
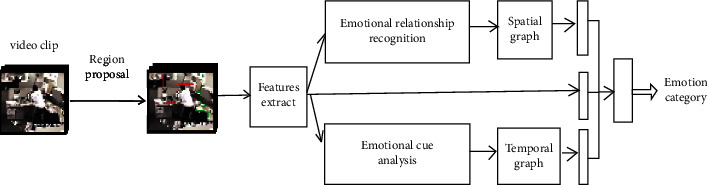
The overall framework of the SE-GCN.

The overall framework of the SE-GCN is shown in Figure 2. It takes as input one video clip which is sampled into F frames for efficiency. To balance efficiency and effectiveness, N emotional elements are selected for each video frame to construct a spatial-temporal graph. Firstly, the region proposal network (RPN) is used to extract the emotional elements contained in the sampled F video frames. Similar to the feature extraction method in [14], VGG-16 is used as the backbone network of faster-RCNN [13], and the FC layer is applied to the emotion proposal regions to obtain the *d*-dimensional appearance feature of each proposal region. Secondly, the sparse spatial-temporal emotion graph is constructed according to these original features of the emotion proposal regions. For the spatial graph, the emotional relationship recognition module is first used to calculate the emotional relationship between any two emotion proposal regions. The sparse spatial graph is constructed according to the emotional relationship. For the temporal graph, the emotional information contained in each emotion proposal region is first analyzed by using the emotional cue analysis module, and the sparse temporal graph is constructed by using the emotion proposal region with rich emotional cues. Then, the spatial-temporal GCN is used to reason about the emotional relationship between emotion proposal regions, and the reasoning features of the emotion relationship are obtained. Finally, the features of the emotion proposal regions and the spatial-temporal relationship features are fused to recognize the video emotion.

### 3.2. Emotional Relationship-Based Sparse Spatial Graph

To construct an emotional relationship-based sparse spatial graph, it is necessary to judge which proposal regions have a closer emotional relationship when emotion proposal regions are extracted. Therefore, we give an emotional relationship recognition module to calculate the emotional relationship between any two proposal regions. After the emotional relationship between proposal regions is obtained, only two proposal regions with close emotional relationships are selected as an edge in the sparse spatial graph. Next, the construction of the emotional relationship-based sparse spatial graph will be described in detail.

The emotional relationship can be expressed as region to region sparse undirected graph G: G=<*V*, *E*>, where each node in V is a proposal region, and each edge e_ij_ ∈ *E*represents the emotional relationship between two nodes. The nodes in the graph can be represented as a set of appearance features of proposal regions *V*={*x*_*i*_*|i*=1,2, ..., *N*}, where N is the number of proposal regions, *x*_*i*_ ∈ *R*^*d*^ is the appearance feature of the *i*th proposal region. We will build *E* ∈ *R*^*N*×*N*^ to determine the node neighborhood.

To reduce the redundant messages and the amount of computation caused by redundant edges and focus on the most relevant relationship to improve the accuracy of emotion recognition, an emotion relationship recognition module is constructed. It takes as input the emotional features of the selected proposal regions and generates the emotional relationship of any two bounding boxes.

Let N be the number of the proposal regions, the emotional features of the proposal regions can be expressed by a matrix *X*, and *X* can be expressed as

(1) $$\begin{array}{c} X=\left(X_{1},X_{2},\ldots X_{N}\right)\text{.} \end{array}$$

It should be noted that the features of proposal regions are disordered. To ensure that the N feature vectors contain face features, the face bounding box is put into VGG-16 and the *d*-dimensional feature vector is extracted. It will be put in the first place on the N × *D* features' vector. Since the GCN layer will not change the order of nodes, we can extract the corresponding face feature.

The emotional feature *X* is input into two fully connected layers and two vectors A and B are obtained, where A and B are *N* × 1 matrices. Multiply the matrices A and the transpose of matrices B to obtain matrix S. Then, a softmax layer is used to calculate the emotional relationship matrix of proposal regions, and it can be expressed as follows:

(2) $$\begin{array}{c} s_{ij}=\frac{\exp\left(s_{ij}\right)}{\sum_{i=1}^{N}\sum_{j=1}^{N}\exp\left(s_{ij}\right)}\text{.} \end{array}$$

After obtaining the emotional relationship matrix between proposal regions, a sparse spatial graph is constructed based on the emotional relationship matrix. For node *i*, the edge <*i*,*j*> is selected as one edge in the sparse spatial graph:

(3) $$\begin{array}{c} \left\{\begin{array}{cc} N_{ij}=1, & s_{ij}\geq T_{iors_{ij}=\max_{1\leq j\leq B}s_{ij}},\\ N_{ij}=0, & else, \end{array}\right. \end{array}$$

where *T*_*i*_ is the threshold. In the experiment, we choose *T*_*i*_ as the mean value of *s*_*ij*_. According to formula (3), we select the edge whose value is greater than the threshold or the maximum value of all vertices connected to it, ensuring that each vertex has at least one edge.

### 3.3. Emotional Information-Based Sparse Temporal Graph

The amount of emotional information contained in emotional proposal regions such as the face, object, and scene is different. Some proposal regions contain rich emotional clues, and the time evolution of these proposal regions can provide rich emotional clues. Some proposal regions contain few emotional cues, and passing messages between these proposal regions will produce a large number of redundant messages. To solve this problem, a sparse temporal graph based on the amount of emotional information is constructed, which only selects the proposal regions with rich emotional clues. Next, we will describe in detail the construction of the sparse temporal graph.

The temporal features of the video can be represented by a sparse undirected graph *G*_*T*_=<*V*_*T*_, *E*_*T*_>. The indexes of rows and columns correspond to the emotional proposal regions in the video and their time order, respectively. *V*_T_ is the set of nodes, which is the appearance features of emotion proposal regions. Each edge e_ij_^T^ ∈ *E*^*T*^ represents the relationship of one emotional proposal region in two adjacent video frames. The indexes are arranged in the time order of the nodes in the video so that the time information is implicitly embedded into the constructed graph. The nodes in the graph can be represented as a set of appearance features of emotion proposal regions in video frames V^*T*^={*x*_*fi*_*|i*=1,2, ..., *N*}, where N is the number of emotion proposal regions, *f*=1,2, ...*F* represents the index of the selected video frame, and *x*_*fi*_ ∈ *R*^*d*^ is the appearance feature of the *i*th emotion proposal region in the *f*th video frame. We will construct *E*^T^ ∈ *R*^*N*×*N*^ to determine the node neighborhood representing the temporal features of emotional proposal regions in the video.

To select the proposal regions with rich emotional clues, we construct an emotional clue analysis module to analyze the amount of emotional information contained in each proposal region. The emotional feature matrix X_*f*_={*x*_*fi*_*|i*=1,2, ..., *N*} represents the emotional feature of the proposal regions in the *f*th video, where *f*=1,2, ...*F* represents the index of the video frame and N is the number of emotional proposal regions in each video frame. *X*_*f*_ is input into a fully connected layer to generate a new feature vector M_*f*_, which is a N × 1 vector. The softmax layer is used to calculate the emotion score of each proposal region. The calculation formula can be expressed as

(4) $$\begin{array}{c} y_{fi}=\frac{\exp\left(M_{fi}\right)}{\sum_{j=1}^{N}\exp\left(M_{fj}\right)}\text{.} \end{array}$$

After obtaining the emotion score of the emotion proposal region of all video frames, a sparse temporal graph can be constructed based on the emotion score. The construction method is as follows:

**Step 1 .**

for the *f*th video frame, the proposal regions with rich emotional information are first selected. The selection method can be expressed as follows:

(5) $$\begin{array}{c} \left\{\begin{array}{cc} X_{fi}isselected, & ify_{fi}\geq T_{f,}\\ X_{fi}\;is\;not\;selected, & else, \end{array}\right. \end{array}$$

where T_f_ represents the threshold of the *f*th video frame and T_f_ selects the intermediate value of *y*_*fi*_ in the experimental stage.

**Step 2 .**

after selecting the proposal regions of each video frame, the sparse temporal graph is constructed. Firstly, the proposal regions with rich emotional clues are selected, and then, those emotion proposal regions with rich emotional clues that appear in adjacent frames are selected. To effectively match the same proposal region in different video frames, the feature similarity of two proposal regions in adjacent frames is measured. Therefore, the adjacency matrix can be expressed as

(6) $$\begin{array}{c} A_{t}\left(t,\left(t+1\right)\right)=\left\{\begin{array}{cc} R\left(x_{ti},x_{\left(t+1\right)i}\right) & dist\left(x_{ti},x_{\left(t+1\right)i}\right)\leq r\\ 0, & else, \end{array}\right. \end{array}$$

where dist(*x*_*ti*_, *x*_(*t*+1)*i*_)=1 − *f*(*x*_*ti*_ ^*T*^*f*(*x*_(*t*+1)*i*_)/‖*f*(*x*_*ti*_)‖ × ‖*f*(*x*_(*t*+1)*i*_)‖ represents the cosine distance between *x*_*ti*_ and *x*_(*t*+1)*i*_, *τ* is a super parameter, and *R*(*x*_*ti*_, *x*_(*t*+1)*i*_) represents the emotional relationship between nodes *x*_*ti*_ and *x*_(*t*+1)*i*_ which can be calculated by the following formula:

(7) $$\begin{array}{c} R\left(x_{ti},x_{\left(t+1\right)i}\right)={\theta \left(f_{ti}\right)}^{T}\phi \left(f_{\left(t+1\right)i}\right)\text{,} \end{array}$$

where *θ*(*x*_*ti*_)=*W*_*θ*_*x*_*ti*_+*b*_*θ*_ and *ϕ*(*x*_*ti*_)=*W*_*ϕ*_*x*_*ti*_+*b*_*ϕ*_ are two learnable linear transformations and *W*_*θ*_, *W*_*ϕ*_, *b*_*θ*_, *b*_*ϕ*_ are weight parameters, which can be obtained through the backpropagation process in the training phase.

### 3.4. Relational Reasoning

The graph convolution network (GCN) reasons the relations by performing message propagation from nodes to their neighbors in the graph. Therefore, once the emotion relation graph is established, we can apply GCN to reason about emotion relations to obtain the features of emotion relation reasoning. Given a graph with N nodes, where each node has a *d*-dimensional feature vector; the operation of the graph convolution can be expressed as

(8) $$\begin{array}{c} X^{\left(l+1\right)}=\sigma \left({\tilde{D}}^{-1/2}\tilde{A}{\tilde{D}}^{-1/2}X^{\left(l\right)}W^{\left(l\right)}\right)\text{,} \end{array}$$

where $\tilde{A}\notin R^{N\times N}$ is the adjacency matrix of the graph, $\tilde{D}\notin R^{N\times N}$ is the degree matrix of $\tilde{A}$, X^(*l*)^ ∉ *R*^*N*×*d*^ is the output of the *l* − 1 layer, W^(*l*)^ is the parameter which can be learned during training, *σ*(·) is nonlinear activation function, and ReLU function is used in this study. In our emotion relationship reasoning framework, the adjacency matrix of the spatial graph is defined in Section 3.2. The adjacency matrix of the temporal graph is defined in Section 3.3. The final output of GCN is the updated features of the nodes in the graph, which can be aggregated into video-level feature vectors for emotional prediction.

### 3.5. Features' Fusion

The features of the emotion proposal regions and the spatial-temporal relationship features are fused to recognize the video emotion. The features of proposal regions X=(*X*_1_, *X*_2_,…, *X*_*N*_) are fused according to the emotion scores Y=(*Y*_1_, *Y*_2_,…, *Y*_*N*_) which are obtained in Section 3.3. The fusion method can be expressed as follows:

(9) $$\begin{array}{c} f_{F=}X\times Y^{T}\text{,} \end{array}$$

where *f*_*F*_ is the fusion feature of proposal regions. It is passed through a full connection layer and obtains a *d*-dimensional feature vector. Thus, the emotional features of F video frames can be expressed as a feature vector *F* × *D*.

After applying GCN on sparse spatial graphs, the spatial relationship reasoning features of the video frames *f*_*G*_=(*f*_*G*_^1^, *f*_*G*_^2^,…, *f*_*G*_^*F*^) can be obtained, where *f*_*G*_^*i*^ is the spatial relationship reasoning features of the *ith* video frames, and it is a *d*-dimensional feature vector. Similarly, after applying GCN on the sparse temporal graph, the temporal relationship reasoning feature of the video *f*_*T*_ is obtained.

To effectively fuse the features of proposal regions, the spatial relationship reasoning features of video frames, and the temporal relationship reasoning feature of the video, *f*_*F*_ and *f*_*G*_=(*f*_*G*_^1^, *f*_*G*_^2^,…, *f*_*G*_^*F*^) are first passed through a full connection layer and obtain a *d*-dimensional feature vector. Secondly, the features of proposal regions, the spatial relationship reasoning features of video frames, and the temporal relationship reasoning feature of the video input into an attention module and obtain the emotional score of each emotional feature. Finally, these features are aggregated according to their emotion score. The aggregated features are sent to the classifier for emotion classification.

## 4. Experiments' Evaluation

### 4.1. Experimental Setup

To evaluate the performance of the proposed method, we conduct experiments on four publicly available video emotion recognition datasets, namely, the MHED dataset [35], the HEIV dataset [36], the VideoEmotion-8 dataset [37], and the Ekman-6 dataset [20].

  MHED [35]: the MHED dataset is composed of 1066 videos, which uses a training set of 638 videos and a testing set of 428 videos. It uses six emotion categories “anger,” “disgust,” “fear,” “joy,” “sadness,” and “surprise,” defined by the psychologists Ekman and Friesen [38] to label human emotions in the video.
  HEIV [36]: the HEIV dataset is composed of 1012 videos with 607 training videos and 405 for testing. Six emotion categories, “anger,” “disgust,” “fear,” “joy,” “sadness,” and “surprise,” defined by the psychologists Ekman and Friesen [38], as well as neutral emotion, are used to label human emotions in the video.
  VideoEmotion-8 [37]: the VideoEmotion-8 dataset contains 1101 videos collected from YouTube and Flickr. The average duration of videos is 107 seconds. These videos are labeled with eight basic emotion categories defined by Plutchik's theory. Similar to [37], ten experiments were carried out. In each training, we randomly select 2/3 of the data for training, and the rest is used for testing. The recognition performance is evaluated by calculating the average accuracy of 10 experiments.
  Ekman-6 [20]: the Ekman-6 dataset contains 1637 videos, of which 819 videos are used for training and 818 videos are used for testing. It is manually annotated by 10 annotators according to Ekman's theory [38] on six basic human emotion categories. There were at least 221 videos in each category, with an average duration of 112 seconds.

### 4.2. Implementation Details

The input video is uniformly partitioned into 20 segments, in which one frame is randomly sampled to obtain 20 frames for one video. For each sampling frame, VGG-16 is used as the backbone network of faster-RCNN [39] to extract the emotional features of proposal regions, and 8 proposal regions are extracted in each video frame. The dimension of each emotional feature is 1024.

In our framework, the sparse spatial graph and sparse temporal graph are trained separately. In the experiment, the network adopts a 6-layer GCN structure. The network is trained in 150 epochs using the minibatch size of 32. The learning rate starts from 0.001 and multiplies to 0.1 every 30 epochs.

### 4.3. Ablation Study

In this section, we will conduct detailed ablation studies on the MEHD and the HEIV datasets to understand the impact of different modules on the accuracy of emotion recognition.

#### 4.3.1. Spatial Relationships

Firstly, we study the influence of the spatial relationship of emotional elements on the accuracy of emotion recognition. Taking the features in the emotion context [11] as the benchmark network, we compare the performance of the spatial relationship of emotional proposal regions with the emotion context [11].  Table 1 gives the results. We note that modeling the spatial relationship between emotional elements can improve the performance of emotion recognition. Using sparse GCN to model the spatial relationship between emotional elements can improve the performance of emotion recognition by 1.7% on the MEHD dataset and 1.62% on the HEIV dataset, which verifies the effectiveness of constructing sparse spatial relationships.

#### 4.3.2. Temporal Relationships

We also study the influence of the temporal relationship of emotional elements on the accuracy of emotion recognition. We compare the performance of the temporal relationship of emotional proposal regions with the emotion context [11]. From Table 1, we notice that modeling the temporal relationship between emotional elements can improve the performance of emotion recognition. Using sparse temporal GCN to model the temporal relationship between emotional elements can improve the performance of emotion recognition by 11.69% on the MEHD dataset and 8.89% on the HEIV dataset, which verifies the effectiveness of constructing a sparse temporal relationship.

#### 4.3.3. Spatial-Temporal Relationships

We further study the influence of the spatial-temporal relationship of emotional elements on the accuracy of emotion recognition. The features of the sparse spatial relationship and the sparse temporal relationship are fused. The performance that uses spatial-temporal relationships has higher accuracy than using spatial relationships alone, with performance improved by 1.61% on the MEHD dataset and 0.99% on the HEIV dataset. The performance that uses spatial-temporal relationships also has higher accuracy than using temporal relationships alone, with performance improved by 1.73% on the MEHD dataset and 1.82% on the HEIV dataset.

#### 4.3.4. Emotion Features of Proposal Regions

We further add the module of emotion features of proposal regions. From Table 2, we can see that the recognition performance improves by 0.7% on the MEHD dataset and 0.49% on the HEIV dataset.

#### 4.3.5. The Influence of Parameters

We also study the influence of super parameters on emotion recognition performance. We compare the performance of the model when the number of video frames *F* = 5, 10, 15, and 20.  Table 3 shows the recognition accuracy increases with the growth of input frames. This demonstrates that our sparse spatial-temporal graphs not only exploit more useful information from more frames but also are robust to the noise from extra data.

We also compare the performance of spatial relationships when the number of emotional proposal regions, *N* = 2, 4, 6, 8, 10.  Table 4 shows the accuracy of spatial relationship reasoning changes with the increase of the number of emotional proposal regions. The accuracy of spatial relationship increases when N increases from 2 to 8, but the accuracy of spatial relationship reduces when *N* increases from 8 to 10. This shows that increasing the number of emotional elements will not always improve accuracy.

### 4.4. Performance Comparison

To demonstrate the advantage of the proposed approach, we compare the performance of our proposed method with the existing methods on the MEHD and HEIV datasets. The comparison results are given in Table 5. The performance of multimodal features' fusion literature [11] and spatial-temporal feature fusion networks [40] is relatively low. This is because the multimodal features' fusion network only uses multiple modes features, and it does not use attention mechanisms and relational reasoning. The attention clusters [41] are used to extract the fc6 layer features of faces, scenes, and images of the video, and then, they are sent to an attention network. The accuracy of the attention cluster on the MHED and the HEIV datasets is 59.81% and 49.63%, respectively. The performance of the attention clusters is better than those feature fusion methods without an attention mechanism. The HAMF uses a hierarchical attention mechanism to solve the difference of contained emotional information in different modes and different images. The hierarchical attention mechanism further improves the performance. Note that our work attains superior performance for two reasons. Firstly, the sparse spatial emotion graph focuses on the most relevant relationship to improve the accuracy of emotion recognition. Secondly, the sparse temporal emotion graph focuses on the time evolution of emotion-rich clues and reduces the amount of computation and redundant information.

### 4.5. Result on Ekman-6 and VideoEmotion-8

In this section, we conduct experiments on the Ekman-6 [20] and the VideoEmotion-8 [37] datasets to further evaluate the effectiveness of our method. The comparison results are given in Table 6. As shown in Table 6, our sparse spatial-temporal graph method achieves a 1.83% top-1 performance gain on the Ekman-6 dataset and a 1.68% top-1 performance gain on the VideoEmotion-8 dataset, respectively. The emotion in context fuses the features of multiple modes without attention mechanism and relationship reasoning. Thus, its performance is relatively low. The performance in the study by Xu et al. [20] is higher than that of the emotion in context. In [20], an unsupervised method was proposed using the deep Boltzmann machine for learning video representations. It depends on large auxiliary images and video datasets for unsupervised training. In addition, a computationally extensive optimization procedure is required to train the model. The kernelized feature [24] applies the sparse representation to reduce the impact of noise contained in videos, which helps contribute to performance improvement. The concept selection [42] method is constructed to improve the performance by filtering out the nondiscriminate object, scene, and action concepts based on Bayes' theory. Only with a few concepts, it can achieve promising results. Our work constructs the sparse spatiotemporal graph to reduce the impact of noise and reduce the computation.

## 5. Conclusions and Future Work

In this study, we propose a sparse spatial-temporal emotion graph convolutional network (SE-GCN)-based video emotion recognition method. Specifically, SE-GCN is composed of two subnetworks. One is the sparse spatial graph, which is constructed with the help of the emotional relationship recognition module, which can obtain the emotional relationship between any two emotion proposal regions. The other is a sparse temporal graph, which is constructed by using the emotion proposal regions with rich emotional cues. The emotional information contained in each emotion proposal region can be obtained by the emotional cue analysis module. The reasoning features of the emotional relationship are obtained by the spatial-temporal GCN. Finally, the features of the emotion proposal regions and the spatial-temporal relationship features are fused to recognize the video emotion. The experimental results show that SE-GCN can effectively improve the performance of emotion recognition. Next, the proposed method will be evaluated for other video classification tasks, such as action recognition and event detection.

## Figures and Tables

**Table 1:** Exploration of spatial-temporal relationships.

Method	MEHD (%)	HEIV (%)
Base model	55.60	46.17
Sparse spatial graph	65.65	54.07
Sparse temporal graph	64.95	53.33
Spatiotemporal graph	67.29	55.06

**Table 2:** Exploration of emotion features of proposal regions.

Method	MEHD (%)	HEIV (%)
Spatiotemporal graph	67.29	55.06
Spatiotemporal graph + proposal regions' features	67.99	55.55

**Table 3:** Exploration of the number of frames.

The number of frames	5 (%)	10 (%)	15 (%)	20 (%)
MEHD	65.65	66.36	66.82	67.29
HEIV	53.33	54.07	54.56	55.06

**Table 4:** Exploration of the number of proposal regions.

N	2	4 (%)	6 (%)	8 (%)	10 (%)
MEHD	49.31%	50.56	50.88	67.29	50.82
HEIV	53.83%	54.07	54.57	55.06	54.81

**Table 5:** Top-1 accuracy (%) compared with related works on the MHED and HEIV.

Method	Accuracy on MEHD (%)	Accuracy on HEIV (%)
Vielzeuf et al. [40]	53.73	45.93
Chen et al. [11]	55.60	46.17
Attention clusters [41]	59.81	49.63
HAMF	63.08	52.34
Our method	65.89	53.09

**Table 6:** Top-1 accuracy (%) compared with state-of-the-art methods on Ekman-6 and VideoEmotion-8.

Method	Ekman	VideoEmotion-8
Emotion in context [11]	51.8	50.6
Xu et al. [20]	50.4	46.7
Kernelized feature [24]	54.4	49.7
Concept selection [42]	54.40	50.82
Ours	56.23	52.5

## Data Availability

Ekman-6 and VideoEmotion-8 are two public datasets. MHED and HEIV datasets can be obtained from the corresponding author upon request.
